# Polyelectrolyte Complexation of Oligonucleotides by Charged Hydrophobic—Neutral Hydrophilic Block Copolymers

**DOI:** 10.3390/polym11010083

**Published:** 2019-01-07

**Authors:** Alexander E. Marras, Jeffrey R. Vieregg, Jeffrey M. Ting, Jack D. Rubien, Matthew V. Tirrell

**Affiliations:** 1 Institute for Molecular Engineering, University of Chicago, Chicago, IL 60637, USA; marras@uchicago.edu (A.E.M.); jting1@uchicago.edu (J.M.T.); mtirrell@uchicago.edu (M.V.T.); 2 Institute for Molecular Engineering at Argonne National Laboratory, Lemont, IL 60439, USA; 3 Departments of Biology and Physics, Swarthmore College, Swarthmore, PA 19081, USA; jrubien@uchicago.edu

**Keywords:** polyelectrolytes, complex coacervation, oligonucleotides, phase separation, nanoparticles

## Abstract

Polyelectrolyte complex micelles (PCMs, core-shell nanoparticles formed by complexation of a polyelectrolyte with a polyelectrolyte-hydrophilic neutral block copolymer) offer a solution to the critical problem of delivering therapeutic nucleic acids, Despite this, few systematic studies have been conducted on how parameters such as polycation charge density, hydrophobicity, and choice of charged group influence PCM properties, despite evidence that these strongly influence the complexation behavior of polyelectrolyte homopolymers. In this article, we report a comparison of oligonucleotide PCMs and polyelectrolyte complexes formed by poly(lysine) and poly((vinylbenzyl) trimethylammonium) (PVBTMA), a styrenic polycation with comparatively higher charge density, increased hydrophobicity, and a permanent positive charge. All of these differences have been individually suggested to provide increased complex stability, but we find that PVBTMA in fact complexes oligonucleotides more weakly than does poly(lysine), as measured by stability versus added salt. Using small angle X-ray scattering and electron microscopy, we find that PCMs formed from both cationic blocks exhibit very similar structure-property relationships, with PCM radius determined by the cationic block size and shape controlled by the hybridization state of the oligonucleotides. These observations narrow the design space for optimizing therapeutic PCMs and provide new insights into the rich polymer physics of polyelectrolyte self-assembly.

## 1. Introduction

Developing effective non-viral methods for delivery of nucleic acids and other macromolecular therapeutics is one of the most pressing challenges for nanomedicine and polymer science [1,2,3,4]. The potential power of engineered nucleic acids as therapeutic agents is severely limited by the difficulty of overcoming the physical and biological barriers to using them as practical drugs. DNA and RNA molecules’ large size, hydrophilicity, and negative charge largely prevent them from crossing cell membranes and promote their rapid clearance from circulation. Exogeneous nucleic acids are also readily degraded by cellular and serum nucleases and are potent activators of the innate immune system. As a result, therapeutic applications to date have required extensive chemical modification and/or encapsulation of the nucleic acids, most commonly by liposomes and other lipid nanoparticles assembled by hydrophobic interactions [5,6,7,8]. These approaches have demonstrated the effectiveness of nucleic acid therapeutics but come with significant drawbacks, including toxicity, immunogenicity, and most particularly, limited biodistribution. In circulation, lipids are rapidly complexed by apolipoproteins and routed to the liver for metabolism. As a result, nucleic acid drugs to date have either been limited to liver targets or delivered locally. This fundamental limitation suggests the need for alternative strategies for nanoparticle self-assembly; one of the most promising being polyelectrolyte complexation.

Polyelectrolyte complexation describes the preference for oppositely-charged macroions to associate with each other in aqueous solution rather than with small counterions, due to their lower translational entropy per unit charge [5]. If the attraction is strong enough, this leads to phase separation despite all components (usually polymers, but also charged particles, as studied by Paul Dubin and others [6]) being individually solvophilic. The resulting polymer-rich phase can either be liquid (complex coacervate) or a solid precipitate, and the factors that determine which one is formed remain largely unknown despite many years of study. We also lack a quantitative ability to predict how molecular properties such as charge density, charge patterning, chirality, hydrophobicity, and hydrogen bonding propensity determine the boundaries of phase separation and the properties of the resulting complex phase. Despite this, complex coacervates and precipitates are widely used in industry and have gained increasing attention as vehicles for drug delivery [5,7].

Nucleic acids are strongly-charged polyanions, and phase-separated complexes have been observed when DNA molecules (ranging in length from as long as entire chromosomes to as short as individual nucleotides) are mixed with cationic polymers [8,9]. Complexation neutralizes the nucleic acids’ charge, and the resulting complexes (sometimes termed ‘polyplexes’) can be internalized by cells via endocytosis. The cationic polymers poly(lysine) and poly(ethyleneimine) are widely used for gene transfection in vitro and are effective, although toxicity and immunogenicity can become a problem as polymer length increases [10,11]. More importantly, however, the resulting complexes lack colloidal stability in circulation, largely limiting them to local applications in vivo.

**Figure 1:**
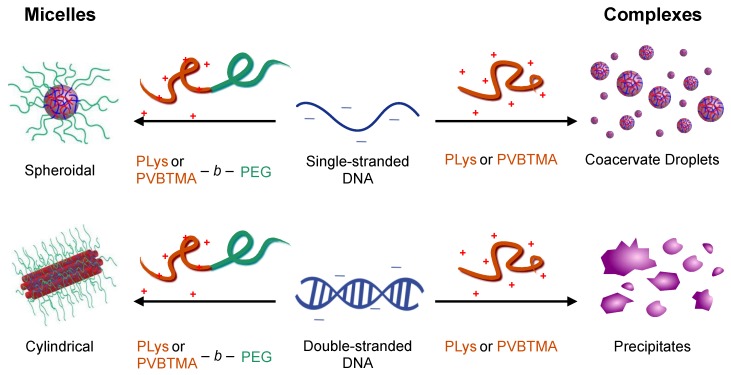
Polyelectrolyte complexation and PCM nanoparticle formation. DNA is a highly charged polyanion. When mixed with polycations (**right**), polyelectrolyte complexes are formed (macrophase separation). When mixed with cation-hydrophilic neutral block copolymers (**left**), microphase separation produces PCMs. In both scenarios, the hybridization state of the nucleic acid (single- vs. double-stranded) determines the nature of the product (liquid droplets vs. solid precipitates for the complexes, spheroidal vs. cylindrical PCMs). This study compares the effect of hydrophilic (poly(lysine), PLys) and hydrophobic (poly((vinylbenzyl) trimethylammonium), PVBTMA) polycations in determining the properties of the complexes and PCMs.

If the polycation is conjugated to a neutral hydrophilic polymer such as poly(ethylene glycol) (PEG), nanoparticles are produced instead of macrophase separation: the hydrophilic neutral block forms a corona around a neutralized polyion core (Figure 1). This is visually reminiscent of surfactant micellization, and the resulting nanoparticles are referred to as polyelectrolyte complex micelles (PCMs, also referred to as polyion complex micelles, block ionomer complexes, and coacervate-core micelles), though the forces driving self-assembly are ionic rather than hydrophobic [12]. Conceptually, PCMs assembled with nucleic acids as the polyanions are attractive delivery vehicles: in addition to the charge neutralization and steric protection from nucleases afforded by the polyion core, the neutral corona provides colloidal stability and size control to allow optimization of circulation properties, as well as a platform for attaching targeting ligands to further improve biodistribution [2]. Assembly of multiple oligonucleotides in each PCM also increases the potency of each cell internalization event, and PCM formation does not require extensive chemical modification of the nucleic acids, preserving biological function. Several promising results in vitro and in small animal models confirm the potential of this strategy [2,13,14,15], but much work remains to optimize PCMs as safe, efficacious nucleic acid delivery vehicles, as well as to improve our understanding of the physics of PCM self-assembly.

We recently investigated structure-property relationships for PCMs formed from DNA oligonucleotides and poly(lysine)-PEG block copolymers, which are by far the most common choice for oligonucleotide delivery [16]. Over a wide range of polymer and block lengths, we found that the PCM core radius is determined solely by the length of the charged block and is independent of both the length and hybridization state (single- vs. double-stranded) of the oligonucleotides. Interestingly, however, we found that oligonucleotide hybridization had a large effect on the shape of the nanoparticles, with single-stranded oligonucleotides forming spheroidal micelles and double-stranded oligonucleotides forming long wormlike micelles, apparently via coaxial stacking of the DNA helices. Small-angle X-ray scattering (SAXS) also revealed parallel packing of DNA helices inside the PCM cores that had previously only been observed for condensed genomic-scale DNA. These results provide design rules for constructing oligonucleotide PCMs of desired size and shape, with exceptionally low polydispersity, but do not address the question of whether poly(lysine) is an optimal cationic polymer to use for oligonucleotide delivery.

**Scheme 1:**
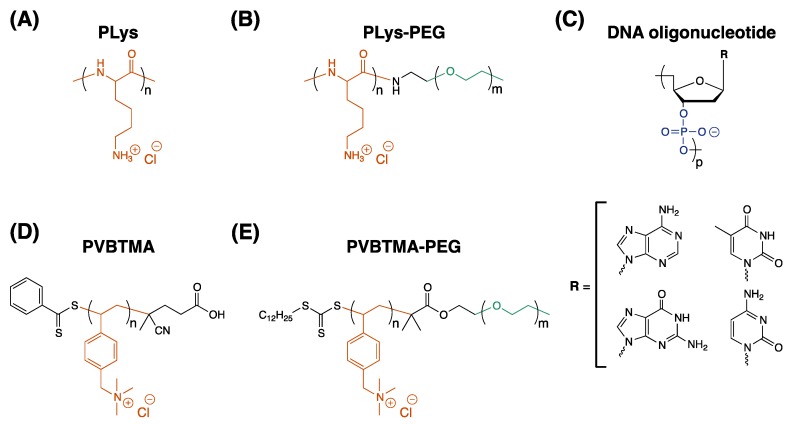
Chemical structures of homopolymers and block copolymers used in this study. PLys (**A**,**B**) is cationic and hydrophilic, where PVBTMA (**D**,**E**) is also cationic but hydrophobic. PEG blocks (**B**,**E**) are neutral and hydrophilic. These polymers complex with DNA oligonucleotides (**C**), which are anionic and hydrophilic, to form phase-separated assemblies.

This study describes an experimental comparison of the styrenic polycation poly((vinylbenzyl) trimethylammonium) and poly(lysine) (PVBTMA and PLys, Scheme 1) as polycations for assembling PCMs and polyelectrolyte complexes with DNA. PVBTMA and PVBTMA-PEG block copolymers are readily accessible via aqueous reversible addition-fragmentation chain transfer (RAFT) polymerization [17] and differ from PLys in several respects that might be expected to influence their complexation behavior. PVBTMA has a higher linear charge density (2 backbone atoms per repeat unit vs. 3), and its aliphatic backbone, aromatic side chain, and minimal propensity for hydrogen bonding make it much more hydrophobic than PLys. PVBTMA’s quaternary ammonium is also permanently charged compared to the primary amine of PLys, which could potentially be deprotonated in the dense environment of the complexes. These differences make for a stringent test of the universality of the design rules we derived for PLys-PEG oligonucleotide PCMs. Additionally, all of these factors have been linked to increased complex stability, which might be expected to improve nuclease resistance and circulation time in the therapeutic setting [18]. As in our previous work, we utilize a multi-modal characterization strategy in which light scattering, SAXS, and light and electron microscopy are used together to provide a more complete picture of the complexes and PCMs than could be obtained from any individual technique.

## 2. Materials and Methods

### 2.1. RAFT Synthesis of PVBTMA and PVBTMA-PEG

PVBTMA homopolymers and PVBTMA-PEG block copolymers were synthesized by aqueous reversible addition-fragmentation chain transfer (RAFT) polymerization as described by Ting et al. [17]. A detailed description is included in Supplementary Materials Section S1. Briefly, PVBTMA homopolymers were synthesized with 35 and 172 repeat units using (vinylbenzyl) trimethylammonium chloride monomer, 4-cyano-4-(phenylcarbonothioylthio) pentanoic acid chain transfer agent (CPhPA, Scheme 1D) and VA-044 thermal initiator in degassed acetate buffer solution/ethanol (3:1 *v*/*v*). PVBTMA-PEG block copolymers were synthesized using trithiocarbonyl macro-CTAs containing PEG blocks of 1k, 5k and 10k MW (Scheme 1E) using the same initiator and buffer. Monomer conversion was assessed by ^1^H NMR spectroscopy; methods and representative spectra are shown in Figures S1–S5. Polymers were extensively dialyzed against NaCl, then water, and lyophilized to provide the chloride salt as a free-flowing powder. Lengths and molecular weights for all the polymers are shown in Table S1.

### 2.2. Polymer Characterization

The absolute molecular weight of the PVBTMA homopolymers and block copolymers was determined by size-exclusion chromatography with multi-angle light scattering (SEC-MALS) using a Waters SEC instrument (Waters Corporation, Milford, CT, USA) equipped with three columns (Waters Ultrahydrogel 500, 250, 120), a diode array detector (Waters 2998), an Optilab T-rEX (Wyatt Technology, Santa Barbara, CA, USA) refractive index detector, and a miniDAWN TREOS II (Wyatt). For all the polymers except the PVBTMA(8)-PEG(5k), the mobile phase was acetonitrile:water (39.9:60 *v*/*v*) with 0.1% trifluoroacetic acid and the flow rate was 1.0 mL/min. Due to its short length, PVBTMA(8)-PEG(5k) was run in aqueous 0.1 M NaNO_3_ with 0.1% (*v*/*v*) NaN_3_. Polymer d*n*/dc values were determined using an Abbe refractometer (Thermo Scientific, Waltham, MA, USA) with a red light-emitting diode at 25 °C; measured refractive index values at increasing polymer concentrations were collected in triplicate and fitted by linear regression (Figure S3).

### 2.3. DNA and Poly(lysine) Polymers

DNA oligonucleotide sequences (Table S2) were designed for minimal self-complementarity and internal structure formation using the NUPACK software tool [19]. Oligonucleotides were ordered from Integrated DNA Technologies (Skokie, IL, USA) and used without further purification. Sheared salmon sperm DNA (average length 2000 bp) was purchased from Invitrogen (Waltham, MA, USA). DNA solutions were resuspended in water at 20 mM charge concentration (mols phosphate/L) prior to use. PLys and PLys-PEG polymers were purchased from Alamanda Polymers (Huntsville, AL, USA) as chloride salts and were neutralized with NaOH and resuspended in water at 10 mM charge concentration prior to use.

### 2.4. Micelle Preparation

PCMs were prepared using the salt-annealing method described by Lueckheide et al. [16]. Briefly, the polyelectrolytes (2 mM final charge concentration) were mixed in PBS buffer (pH 7.4), then concentrated NaCl solution was added to obtain 1M final concentration (400 μL total solution volume) to dissolve the complexes. The salt concentration was then slowly reduced over 36 h by step dialysis with a 2000 MWCO membrane (Slide-a-lyzer G2, ThermoFisher, Waltham, MA, USA) to a final working concentration of 1× PBS (155 mM NaCl, 1 mM KH_2_PO_4_, 3 mM Na_2_HPO_4_-7H_2_O, pH 7.4).

### 2.5. Small-Angle X-ray Scattering

SAXS measurements were made at beamline 12-ID-B of the Advanced Photon Source at Argonne National Laboratory (Lemont, IL, USA). Micelle samples were irradiated in a thin-wall glass capillary flow cell with a photon energy of 14 keV. Data was reduced and background was subtracted as described in Ref. [16]. Fitting was performed using the multi-level modeling macros distributed with the Irena software package [20] for Igor Pro as described in the same reference.

### 2.6. Electron Microscopy

Cryo Transmission Electron Microscopy (TEM) samples were flash frozen onto lacey carbon film grids (LC200-CU, Electron Microscopy Sciences, Hatflield, PA, USA) and imaged on a FEI Talos TEM (FEI, Hillsboro, OR, USA) at an acceleration voltage of 200kV. Negative-stained samples were deposited on carbon coated square grids (CF200-Cu-UL, Electron Microscopy Sciences), dried, and stained with 2% uranyl formate, and imaged on a FEI Tecnai G2 Spirit TEM at an acceleration voltage of 120 kV.

### 2.7. Micelle Salt Dependence

To assess the stability of the micelles vs. salt, 200 μL micelle samples were prepared as described above and titrated with 5M NaCl in steps of 100 mM. Light scattering intensity was measured at a 90-degree angle using a Brookhaven BI-200SM (Brookhaven Instruments, Holtsville, NY, USA) system with a 637 nm laser at room temperature. Critical salt concentrations were classified as the point where no structure is seen in the autocorrelation function and scattering intensity drops below 5000 counts per second. +/−50 mM error bars are shown to reflect the finite step size used in titration.

### 2.8. Homopolymer Complex Preparation

Polyelectrolyte complexes were prepared at pH 7 and room temperature. Double-stranded DNA was prepared by annealing complementary strands at 65 °C for 5 min followed by slow cooling to RT. 18.2 MΩ water, concentrated PBS, and NaCl (when applicable) solutions were mixed, followed by addition of the DNA and then the polycation for a final concentration of 2 mM charge concentration of polyelectrolytes and 1× PBS. Samples were mixed thoroughly after addition of each polyelectrolyte. Aliquots were prepared separately at NaCl concentrations up to 1400 mM with 100 mM increments.

### 2.9. Optical Microscopy

Phase and morphology of the homopolymer complexes were observed by bright field optical microscopy using a Leica DMI-6000B inverted microscope (Leica Microsystems, Buffalo Grove, IL, USA) with white light illumination and 10–20× magnification. 100 μL aliquots of the complex suspensions were placed in ultra-low attachment 96 well plates (Costar, Corning, Tewksbury, MA, USA). Images were taken shortly after mixing and then again 4 h later, with the latter used unless noted to the contrary.

## 3. Results

### 3.1. Polyelectrolyte Complex Micelle Formation and Morphology

**Figure 2:**
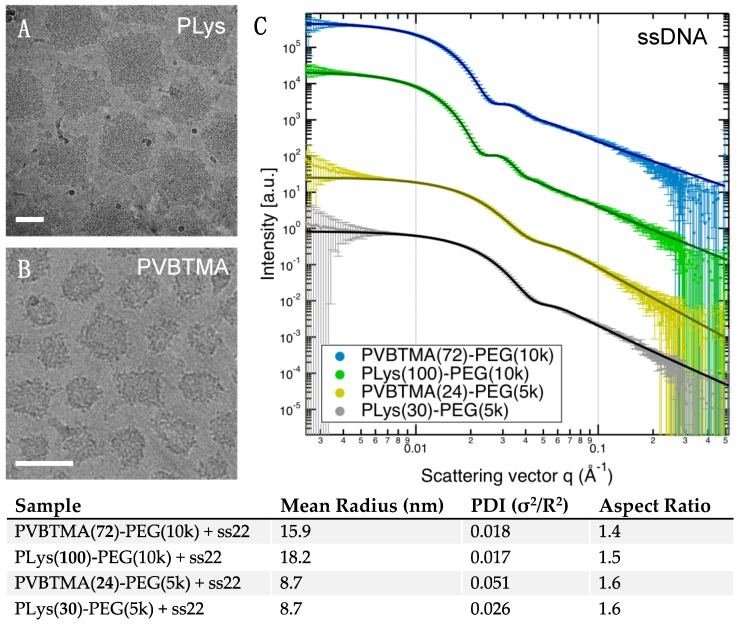
Characterization of single-stranded oligonucleotide polyelectrolyte complex micelles. PLys-PEG and PVBTMA-PEG both form spheroidal micelles with ssDNA in all cases tested. Examples here show cryo electron micrographs of PLys(200)-PEG(10k) with 88 nt DNA (**A**) and PVBTMA(72)-PEG(10k) with 88 nt DNA (**B**). Small-angle X-ray scattering intensity profiles and fits provide further structural information, as shown with four examples in (**C**) with fitting results shown in the table. Fit results for all ssDNA PCMs are shown in Tables S4 and S5. Scattering curves are vertically offset for clarity. Scale bars = 50 nm.

**Figure 3:**
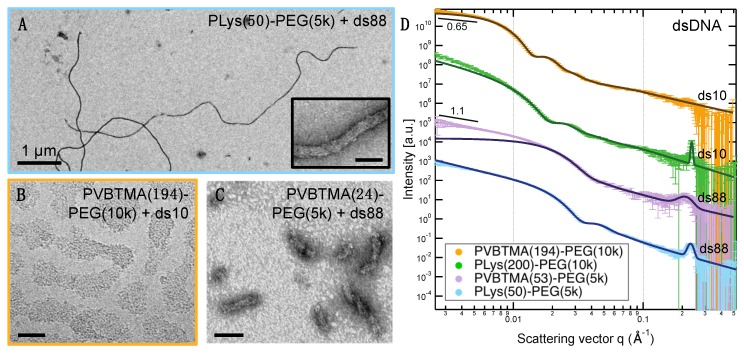
Characterization of double-stranded oligonucleotide polyelectrolyte complex micelles. A discrepancy in length and shape is seen with dsDNA PCMs. PLys-PEG forms long flexible cylinders (**A**), while PVBTMA-PEG forms cylindrical or spheroidal PCMs with polydisperse lengths (**B**,**C**). (**D**) shows representative SAXS data for both systems with fitting results available in Table 1. Panel A is PLys(50)-PEG(5k) + 88 bp DNA, (**B**) is PVBTMA(194)-PEG(10k) + 10 bp DNA, and (**C**) is PVBTMA(24)-PEG(5k) + 88 bp. (**A**,**C**) negative-stain conventional EM and (**B**) is a cryo-EM micrograph. Scale bars = 50 nm unless noted.

In order to evaluate PVBTMA as a cationic polymer for nucleic acid delivery, we first prepared PCMs using PVBTMA-PEG block copolymers and single-stranded DNA (ssDNA) oligonucleotides and compared them to PCMs assembled from PLys-PEG block copolymers of similar lengths using SAXS and electron microscopy (EM). None of the polymers formed detectable structures on their own (Figure S6). The block copolymer with the shortest PVBTMA polycation (PVBTMA(8)-PEG(5k) also did not phase separate when mixed with DNA, and block copolymers with the shortest PEG lengths (1k MW) formed large aggregates rather than nanoparticles. In all other cases, however, both cationic polymers readily formed PCMs when mixed with DNA under these conditions, with low polydispersity in radius (Figure 2 and Figure 3). We were able to accurately fit the SAXS scattering profiles with a combination of hard-surface form factor, power-law, and diffraction peak models (Figure S7). EM imaging (cryo and conventional) also confirms PCM formation with both cationic polymers.

**Figure 4:**
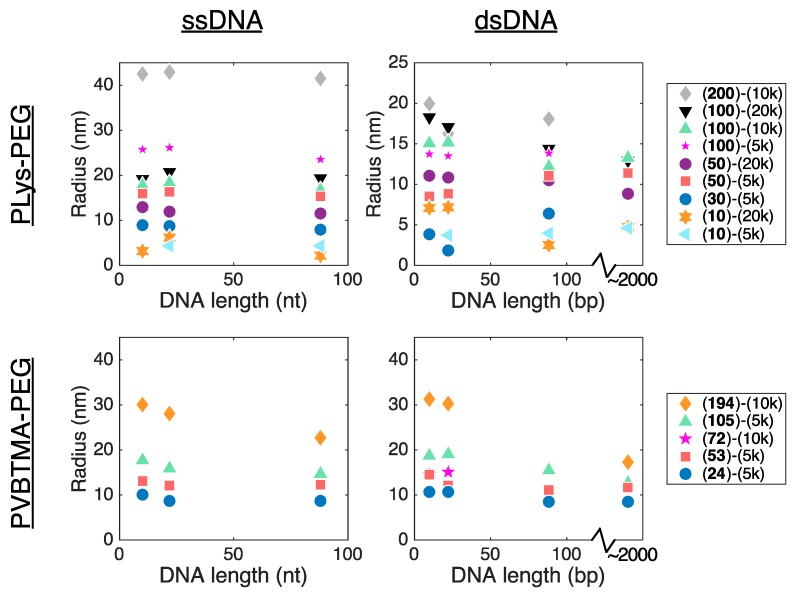
PCM radii from SAXS fits vs. polyelectrolyte length. For both cations, PCM radius is largely insensitive to nucleic acid length but increases strongly with the length of the cation blocks. Data is tabulated in the Supplementary Materials.

The low-q region of the SAXS scattering profiles provides information on PCM size and shape, and numerous similarities are observed for PLys and PVBTMA micelles. With both polymers, spheroidal micelles are observed for PCMs containing single-stranded oligonucleotides, as shown by the flat (q^0^) scattering intensity in the low-q region of the SAXS data and corroborated by cryo-TEM (Figure 2). Fitting shows that the micelle radii are similar for both polymers (Figure 2 and Figure 4). As previously observed with PLys, we saw no dependence of PCM radius on oligonucleotide length over a 9-fold range in the latter, but a marked increase in radius with cationic block length (Figure 4). These results, which extend over a larger range of block lengths than our previous work, suggest that the principles governing PCM formation apply over a wide range of polymer structures and chemical properties. Direct modification of the PVBTMA chain end-groups in otherwise-equivalent systems also impacts the micelle particle size (Figure S8 and Table S3). Additional EM and SAXS data and fits are available in Tables S4–S7 and Figures S9–S19.

For double-stranded DNA (dsDNA), we observe long, wormlike micelles with the PLys-PEG block copolymers (Figure 3A), consistent with our previous report, but in this case we see a qualitative difference for PCMs prepared with PVBTMA-PEG. The micelles exhibit low polydispersity in radius, with fitted values very similar to those found for PLys-PEG PCMs (Table 1), but the lengths are shorter and highly variable between and within samples (Figure 3B,C). This leads to power-law dependencies of the SAXS scattering intensity in the low-q region that are not consistent with spheroidal (q^0^), rigid cylinder (q^1^), or flexible cylinder (q^2^) form factor models (Table 1). Accordingly, we did not attempt to fit the lengths of these micelles and used a sphere form factor model with a low-q cutoff to determine the radius alone (Figure 3D and Figure S19). Just as for single-stranded DNA, we see minimal dependence of micelle radius on DNA length (Figure 4), even for sheared salmon sperm DNA (mean length ~2000 bp), which we included as an analog for plasmids and other full-length gene constructs. Also, similarly to the ssDNA case, we observe a marked increase in PCM radius with charged block length (Table 1, Figure 4).

The higher-q region (q ≥ ~0.08 Å^−1^) of the SAXS data provides information about the internal structure of the micelles. Consistent with our previous observations, the ssDNA PCMs showed, with few exceptions, Porod exponents near 2 regardless of whether PLys or PVBTMA was used as the cationic blocks. This indicates that the polymers are behaving like ideal (i.e., uncharged) chains and is consistent with idea of a liquid-like PCM core for these micelles. For dsDNA PCMs formed with PLys-PEG, we previously showed that a prominent diffraction peak at q ≈ 0.23 Å^−1^ resulted from parallel, hexagonal packing of dsDNA helices within the PCM cores very similar to those observed in toroidal condensates of genomic DNA [16]. Though shifted to lower q and broadened (Figure 3D), a similar diffraction peak is observed for many PVBTMA-PEG PCMs, implying similar, though perhaps less regular, ordering within these micelles.

### 3.2. PCM Stability

**Figure 5:**
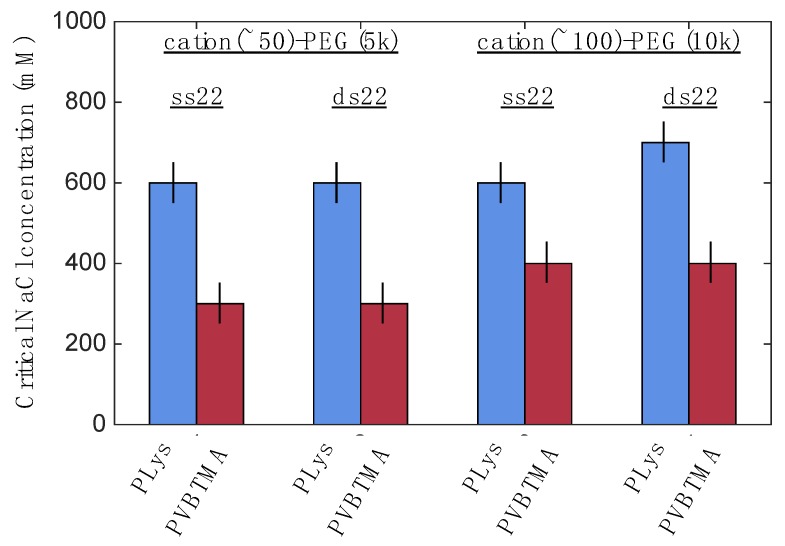
PVBTMA PCMs are much less resistant to added salt compared to PLys PCMs. Critical salt concentration (measured by drop in light scattering) is plotted vs. cation identity and length. Error bars show titration step size (100 mM NaCl).

The primary driving force for polyelectrolyte complexation is entropy gain from counterion release [21]. Increasing the background ionic strength decreases this, eventually resulting in dissolution of the complexes. The critical ionic strength required for dissolution therefore provides a measure of the stability of a given complex [22]. To compare the stability of PVBTMA-PEG vs. PLys-PEG PCMs, we titrated concentrated NaCl into PCMs prepared at physiological ionic strength while monitoring light scattering intensity. As shown in Figure 5, PCMs containing PVBTMA-PEG are considerably less stable than those containing PLys-PEG: PLys-PEG PCMs are stable up to 600–700 mM NaCl compared to PVBTMA-PEG PCMs which no longer form micelles above 300–400 mM.

### 3.3. Homopolymer Complexation

**Figure 6:**
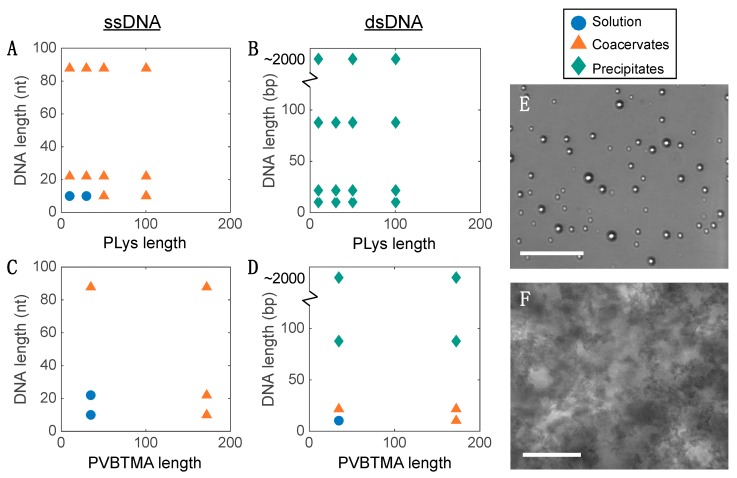
Phase diagrams for ssDNA (**A**,**C**) and dsDNA (**B**,**D**) complexing with homopolymers PLys (**A**,**B**) and PVBTMA (**C**,**D**). PVBTMA requires longer oligonucleotides for phase separation to occur, suggesting a less stable interaction with DNA. (**E**) Representative optical micrograph of coacervate complexes (22-nt ssDNA + 50-aa PLys). (**F**) Representative optical micrograph of precipitate complexes (88-bp dsDNA + 53mer PVBTMA). Scale bars = 50 µm.

In order to more fully understand the complexation behavior of PVBTMA and DNA, we next compared complexes formed by PVBTMA/PLys homopolymers (no neutral block) and DNA oligonucleotides over a similar range of lengths as used in the PCM studies. We have previously observed that, under these conditions, PLys and DNA readily phase separate, forming either liquid droplets (coacervates) or solid precipitates depending on whether the DNA is single- or double-stranded [23]. As shown in Figure 6, PVBTMA shows the same phase behavior when complexed with long DNA (88 nt/bp and above), but is significantly less effective at forming phase-separated complexes with shorter oligonucleotides. (35)mer PVBTMA does not phase separate at all with 22 nt ssDNA, and both (35) and (174)mer PVBTMA form coacervates rather than precipitates when mixed with shorter double-stranded oligonucleotides. Overall, PVBTMA requires longer polyelectrolyte lengths for phase separation to occur compared to PLys.

**Figure 7:**
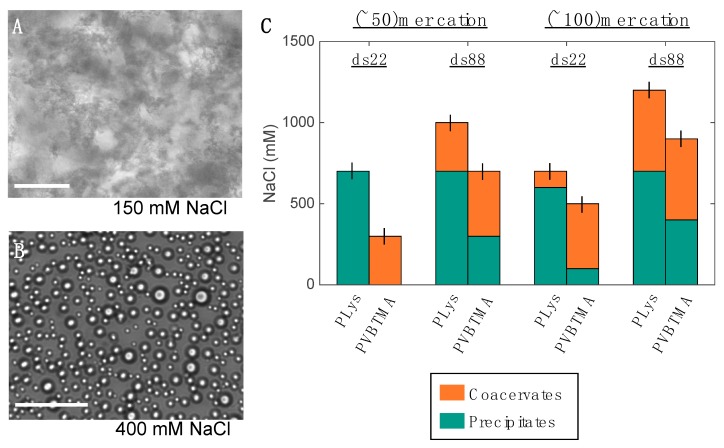
Phase transitions of homopolymer complexes with increasing salt concentration. As ionic strength increases, precipitates ((**A**), PVBTMA(35) – dsDNA(22)) melt into coacervate droplets (**B**) before eventually dissolving. Critical ionic strengths for melting (green to orange) and dissolution are plotted in (**C**) for four different combinations of polyelectrolyte lengths. At each length combination, phase transitions occur at lower ionic strength for PVBTMA than for PLys. Scale bars = 50 µm.

Finally, we measured the effect of increasing ionic strength on the PVBTMA – DNA homopolymer complexes and compared this to our previous results for PLys – DNA complexes [23]. Qualitatively, we observe similar trends for both polycations (Figure 7). As salt concentration increases, solid precipitates (for double-stranded DNA complexes, representative data for PVBTMA(35) – dsDNA(22) at 150 mM NaCl shown in panel A) melt into coacervates (panel B, 400 mM NaCl), then dissociate into solution. Complexes formed from longer polymers are more stable than those formed from shorter ones. In each case, however, the phase transitions (solid – liquid, liquid – soluble) occur at much lower salt concentrations for PVBTMA than for PLys, with physiological ionic strength (PBS buffer) sufficient to melt the PVBTMA(35) – dsDNA(22) complexes into coacervates. The critical ionic strengths required to dissolve the homopolymer complexes are also quite close to those measured for the PCMs (Figure 5), implying that the geometric constraints imposed by microphase separation do not significantly decrease the stability of the polyelectrolyte complexes in the core.

## 4. Discussion

These results provide, to our knowledge, the first systematic characterization of the ability of PVBTMA to complex nucleic acids; previous studies had been limited to single-angle DLS measurements of complexes made with highly-polydisperse sheared genomic DNA [24]. This reflects a more general trend in the field: with only few exceptions [25,26], investigations of polycations for nucleic acid delivery have focused on hydrophilic polymers such as PLys, poly(ethyleneimine), poly(amidoamine), and cationic polysaccharides [10,12], many of which are analogs of natural products. This may represent a missed opportunity, as synthetic polymers provide access to diverse structural motifs, many of which are bio-orthogonal, and allow tuning of intermolecular interactions (hydrophobicity, hydrogen bonding, cation-pi, etc.) over a wider range than is possible with naturally-inspired polycations. Given the diverse range of interactions available with nucleic acids (charge interactions through the phosphate anion, pi-pi and cation-pi interactions with the nucleobases, hydrogen bonding from the sugar and nucleobase heteroatoms, and more), it seems reasonable to assume that synthetic polymers may provide more optimal vehicles for therapeutic delivery. This diversity, however, raises an important question: to what degree can lessons learned with one polycation be transferred to another? This study was designed to provide a stringent test of that proposition; as discussed earlier, PVBTMA has a 50 percent larger linear charge density than PLys, has an aliphatic backbone and aromatic side chains, is largely unable to form hydrogen bonds, and contains a permanent positive charge rather than the ionizable primary amine.

Remarkably, we find many more similarities than differences when comparing PCMs formed by the two polycations. PLys-PEG and PVBTMA-PEG both form micelles with low polydispersity in radius when prepared via salt annealing, and the PCM radii are, in most cases, quantitatively similar as well. (Figure 2, Figure 3 and Figure 4; Table 1 and Tables S4–S7). We have previously reported that the radius of oligonucleotide PCMs formed with PLys-PEG block copolymers is controlled by the length of the charged block but largely insensitive to oligonucleotide length [16]. These results extend this conclusion to larger block lengths (n ≈ 200) and show that the same pattern is true for PVBTMA-PEG PCMs (Figure 4), suggesting that this may be a universal property for PCMs.

We also observe that, below critical lengths for both the charged and neutral blocks, the complexes aggregate rather than forming nanoparticles. Similar results have been reported for PCMs formed from synthetic acrylate-based polyelectrolytes and block copolymers, though those authors discussed it in terms of core-corona ratio [27]. While this requirement is likely general, determining the exact criteria for colloidal stability and whether a quantitative analog to the ‘packing parameter’ that describes hydrophobic micellization exists for PCMs remains an open question.

We have previously seen that the hybridization state of the nucleic acid is a key parameter in determining the behavior of polyelectrolyte complexes and PCMs assembled using PLys as the polycation [16,23]. Single-stranded oligonucleotides produce liquid complexes and spheroidal micelles, while double-stranded nucleic acids produce solid complexes and long cylindrical micelles with substantial internal ordering in the form of hexagonally-packed and coaxially-stacked DNA helices. With both SAXS and EM, we observe similar, though not identical behavior when PVBTMA is used as the polycation. Single-stranded PVBTMA-PEG PCMs are spheroidal, and double-stranded PCMs form cylinders, but the lengths of the latter are much shorter than seen with PLys and are highly polydisperse. A similar pattern is found for the homopolymers: liquids are observed for ssDNA complexes, and solids are only seen with dsDNA, but the short-polymer region where no phase separation is observed is larger with PVBTMA, and liquids are also observed for complexes containing short (10 and 22 bp) double-stranded oligonucleotides.

One hypothesis that explains these differences is that the interactions between DNA and PVBTMA are simply weaker than those with PLys; this is strongly supported by the NaCl stability data, which show that PCMs and homopolymer complexes containing PVBTMA are significantly easier to melt and/or dissolve with salt than those containing PLys. In this interpretation, the polydispersity in length observed for the dsDNA + PVBTMA-PEG PCMs results from some combination of the more favorable entropy associated with a larger number of shorter PCMs and mechanical instability of the micelles during experimental manipulations. At the molecular level, this length dispersity implies less efficient coaxial stacking of DNA helices, while the observed broadening and shift of the packing diffraction peak to lower q when PLys-PEG is replaced by PVBTMA-PEG reflects less effective helical packing through a decrease in order and an increase in average inter-helix spacing.

While this hypothesis appears to be consistent with our data, it may also seem counterintuitive, as many of the structural differences between PVBTMA and PLys (higher charge density, increased hydrophobicity, non-susceptibility to neutralization) have been individually shown or suggested to increase polyelectrolyte complex stability [28,29,30,31]. We have also observed that PVBTMA and polystyrene sulfonate (PSS) form polyelectrolyte complexes that are extremely stable with respect to salt [32]. A closer look at the structures and potential intermolecular interactions of the polyelectrolytes suggests several solutions to this apparent conflict. One possibility with some experimental evidence is that, despite their permanent charge, the steric bulk of quaternary amines limits their effectiveness for polyelectrolyte complexation by enforcing a larger separation between opposing charges than is attainable with smaller cations and counterions. Schlenoff and coworkers recently reported that poly(allylamine) and poly(vinylamine), which have primary amines as charged groups, formed stronger complexes than did three quaternary amine-containing polycations, including PVBTMA [33]. Similarly, Izumrudov and coworkers found that PLys and several other primary amine-containing polycations formed complexes with sheared genomic DNA that were more resistant to salt than those formed with quaternary amine-containing polycations [34]. Hydrophobic interactions offer additional opportunities for stabilization, but the disparate structures of DNA and PVBTMA may hinder short-range interactions such as pi stacking that are favored in symmetrical polyelectrolytes such as PVBTMA and PSS. Conjugation of hydrophobic moieties to one end of the polyelectrolytes has been shown to increase the serum stability of nucleic acid PCMs [18,35], and the smaller radii we observe in PCMs with PVBTMA-PEG block copolymers containing C_12_ tails (Figure S8) highlights the potential of RAFT polymerization to access these more complex architectures. A final possibility is that PLys-DNA complexes could also be stabilized by hydrogen bonding interactions that are not available to PVBTMA.

While representing only a single pair of polycations, the large structural differences between PLys and PVBTMA suggest that the similarities in PCM properties that we observe may be universal, while the differences provide useful leads for further investigation into the physics of polyelectrolyte self-assembly as well as possible solutions to the pressing problem of nucleic acid delivery. The diversity of structures accessible in synthetic polymers, as well as the capability of modern polymer synthesis techniques such as RAFT to access these in a relatively modular manner, offers exciting new possibilities on both fronts. In particular, systematic variation of charged groups, side chain structures, and backbone architectures should be able to unravel the effects of hydrophobicity, charge density and type, and hydrogen bonding and enable rational design of optimal delivery vehicles for therapeutic nucleic acids.

## Figures and Tables

**Table 1:** Representative SAXS fit parameters for dsDNA PCMs. All PCMs show low polydispersity in radius, but the PVBTMA-PEG PCMs exhibit intermediate scaling laws at low-q indicating significant polydispersity in length. PLys-PEG data are fit using flexible cylinder form factors (plus power law and diffraction peak at higher q) while PVBTMA-PEG data is fit using a sphere form factor cut off at low-q. Fit results for all dsDNA PCMs are shown in Tables S6 and S7.

Sample	Mean Radius (nm)	PDI (σ^2^/R^2^)	Packing Peak	Low-q Power Law
PVBTMA(**194**)-PEG(10k) + ds10	31.3	0.02	No	0.65
PVBTMA(**194**)-PEG(10k) + ds22	30.3	0.04	Yes	1.59
PVBTMA(**53**)-PEG(5k) + ds22	12.1	0.09	Yes	1.92
PVBTMA(**53**)-PEG(5k) + ds88	11.0	0.05	Yes	1.13
PLys(**200**)-PEG(10k) + ds10	19.9	0.03	Yes	
PLys(**200**)-PEG(10k) + ds22	16.3	0.01	Yes	
PLys(**50**)-PEG(5k) + ds22	8.8	0.03	Yes	
PLys(**50**)-PEG(5k) + ds88	11.0	0.03	Yes	

## Supplementary Materials

The following are available online at http://www.mdpi.com/2073-4360/11/1/83/s1: Expanded experimental section, additional EM images, additional scattering data and tabulated fitting parameters. The SAXS data sets are available at https://doi.org/10.18126/M2QW8R.
