# Supplementary material for: Polyelectrolyte Complexation of Oligonucleotides by Charged Hydrophobic—Neutral Hydrophilic Block Copolymers
Source: Polymers (Basel). 2019 Jan 7;11(1):83. doi: 10.3390/polym11010083 (PMC6402004; doi:10.3390/polym11010083)
Supplement: Supplementary file 1 [file polymers-11-00083-s001.pdf]

Polyelectrolyte Complexation of Oligonucleotides by Charged Hydrophobic – Neutral Hydrophilic Block Copolymers

Alexander E. Marras, Jeffrey R. Vieregg, Jeffrey M. Ting,
Jack D. Rubien, and Matthew V. Tirrell

S1. Experimental Information

Materials. The following reagent grade materials were used as received, unless otherwise specified: 4-cyano-4-(phenylcarbonothioylthio)pentanoic acid (CPhPA, Sigma), poly(ethylene glycol) methyl ether (2-methyl-2-propionic acid dodecyl trithiocarbonate) (PEG-C12, Sigma, Reported M_n 1100, 5000, and 10,000 g/mol), poly(ethylene glycol) 4-cyano-4-(phenylcarbonothioylthio)pentanoate (PEG-Sty, Sigma, Reported M_n 10,000 g/mol), (vinylbenzyl)trimethylammonium chloride (VBTMA, Sigma, 99%), 2,2'-azobis[2-(2-imidazolin-2-yl)propane]dihydrochloride (VA-044, Wako Chemicals, USA), 1,6-diphenyl-1,3,5-hexatriene (DPH), acetic acid (glacial, Sigma, ≥99.85%), sodium acetate trihydrate (Sigma, ≥99%), hydrogen peroxide (H_2O_2, Sigma, 30% *w*/*w* in H_2O), ethanol (anhydrous, Decon 200 proof), and SnakeSkin dialysis tubing / Slide-A-Lyzer dialysis cassettes (MWCO 3.5K, 22 mm, Thermo Scientific). Acetate buffer solution was prepared using 0.1 M acetic acid and 0.1 M sodium acetate trihydrate (0.1 M) (42/158, *v*/*v*), adjusted to pH 5.2 for RAFT polymerizations. Unless otherwise stated, all water was used from a Milli-Q water purification system at a resistivity of 18.2 MΩ-cm at 25 °C.

RAFT polymerization synthesis. A full description of the reversible addition-fragmentation chain transfer (RAFT) synthesis can be found in Ting et al.[1] The following PVBTMA-PEG block polyelectrolytes were prepared in a Carousel 12 Plus Reaction Station (Radleys, Saffron Walden, UK): 53-5k, 105-5k, and 72-10k. The 60-1k and 24-5k systems were synthesized in a round bottom flask, while the 8-5k system was quenched at ~10% conversion of a 105-5k, anticipated from previously-conducted kinetics experiments.[1] PVBTMA(**35**) and PVBTMA(**172**) homopolymers were also prepared in the carousel reactor.

In general, to each glass container, the chemical precursors (monomer, RAFT macromolecular PEG chain transfer agent / CPhPA chain transfer agent, and VA-044 initiator) were combined in acetate buffer solution and sealed. 10:1 equivalence of RAFT chain transfer agent to initiator was used. Carousel reactions were degassed simultaneously via three freeze-pump-thaw cycles; a mixture of acetate buffer solution and ethanol (3:1, *v*/*v*) was used for nitrogen-mediated bubbling to degas the round bottom flask reactions. Reactions were run at 50 °C under constant stirring for 21 h, with

the final monomer conversion confirmed by ^{1}H NMR spectroscopy before quenching by cooling to room temperature and opening the reactors to air.

^{1}H NMR Spectroscopy. ^{1}H NMR experiments were conducted on a Bruker AVANCE III HD 400 Mhz NanoBay spectrometer with 16 transients to minimize signal-to-noise. ^{1}H NMR spectra were processed and analyzed using iNMR (Version 5.5.7).

Figure S1 shows a representative ^{1}H NMR spectrum for the PVBTMA(**172**) homopolymer. ^{1}H NMR (400 MHz, D_2O) δ: 1.0-2.5 ppm (alkyl backbone, 3H, –C**H**$_2$-C**H**–), 2.5-3.1 ppm (9H, –N-(C**H**$_3$)$_3$), 4.1-4.5 ppm (2H, –C**H**$_2$-N–), and 6.1-7.3 ppm (4H, Ar**H**).

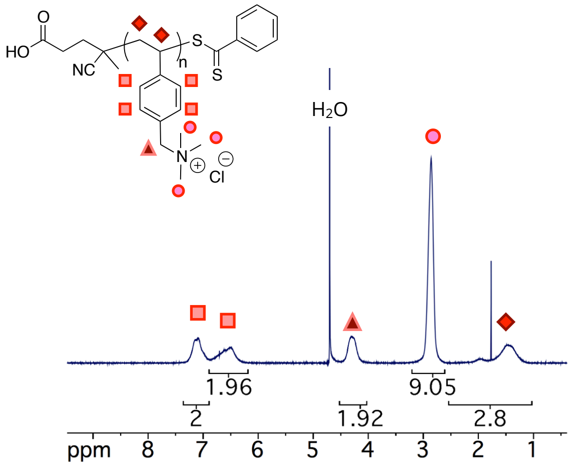

Figure S1. Representative ^{1}H NMR spectrum of PVBTMA(**172**) in D_2O.

Fig. S2 shows a representative ^{1}H NMR spectrum for the PVBTMA(**60**)-PEG(1k) block polymer. ^{1}H NMR (400 MHz, D_2O) δ: 1.1-2.5 ppm (alkyl backbone, 3H, –C**H**$_2$-C**H**–), 2.6-3.0 ppm (9H, –N-(C**H**$_3$)$_3$), 3.4-3.6 ppm (4H, –O-(C**H**$_2$)$_2$–), 4.0-4.5 ppm (2H, –C**H**$_2$-N–), and 6.3-7.3 ppm (4H, Ar**H**).

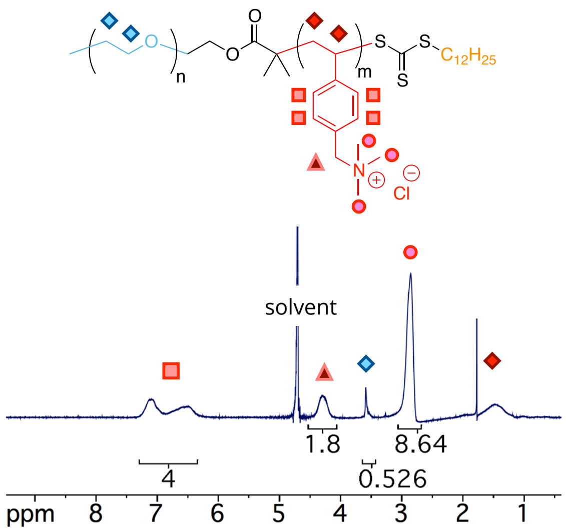

Figure S2. Representative ^{1}H NMR spectrum of PVBTMA(**60**)-PEG(1k) in D_2O.

d*n*/dc Measurements. Fig. S3 shows the data used to determine the change in refractive index (d*n*/dc) values for PVBTMA(**172**). The calculated coefficient of determination (R^2 = 0.99) confirms the linear relationship between refractive index and polymer concentration. Previous d*n*/dc measurements of the PVBTMA-PEG diblock polyelectrolytes can be found in the literature.[1]

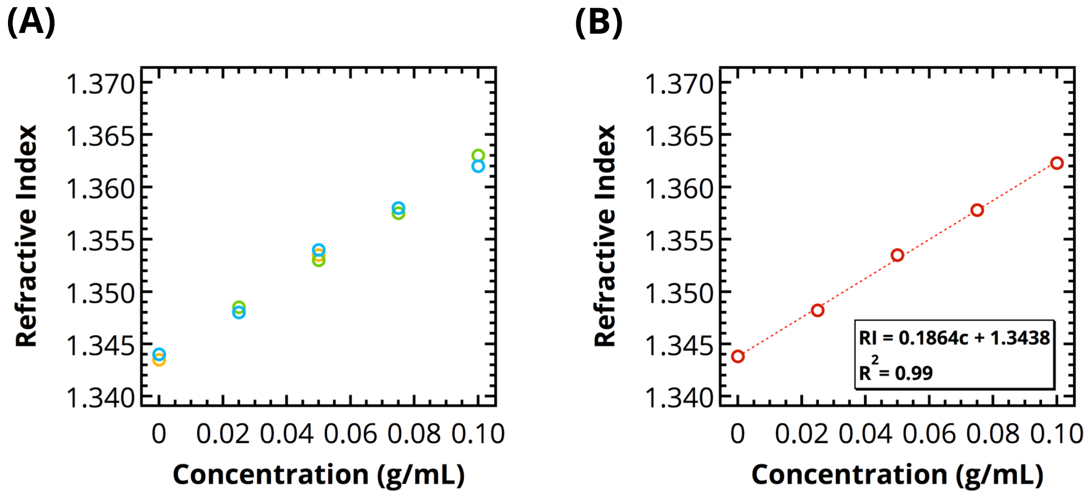

Figure S3. Determination of d*n*/dc for PVBTMA(**172**) in the size-exclusion chromatography mobile phase (60% water, 39.9% acetonitrile, 0.1% trifluoroacetic acid), via (A) measurements taken in triplicate and (B) a linear regression to the average of the data points.

Size Exclusion Chromatography. Fig. S4 shows a representative SEC chromatogram using the refractive index (RI) detector for the PVBTMA(**60**)-PEG(1k) system. All samples outlined in Table S1 below demonstrated a unimodal peak.

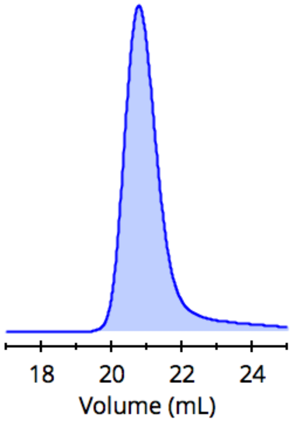

Figure S4. Representative SEC RI chromatogram of the PVBTMA(**60**)-PEG(1k) in a mobile phase of 59.9% water, 40% acetonitrile, and 0.1% trifluoroacetic acid.

RAFT end group removal. The RAFT end-group removal reaction was carried out following protocols reported by Jesson et al.[2] H_2O_2 was used as a mild oxidant to cleave thiocarbonylthio chain ends at a molar ratio of 5:1 (H_2O_2 to chain transfer agent) at 70 °C for 8 h open to the air in water, at 7.5% *w/w*. Visually, the solution changed from light yellow to colorless. Previous reports have shown that PEG can be susceptible to free radical-induced degradation in water when exposed to UV/H_2O_2.[3] Thus, as a precaution reactions were conducted in a dark environment. As seen in Figure S5, the disappearance of the polymer trithiocarbonate peak centered at 310 nm in water by UV-vis spectroscopy confirms successful end-group removal for the PVBTMA(**72**)-PEG(10k) diblock.

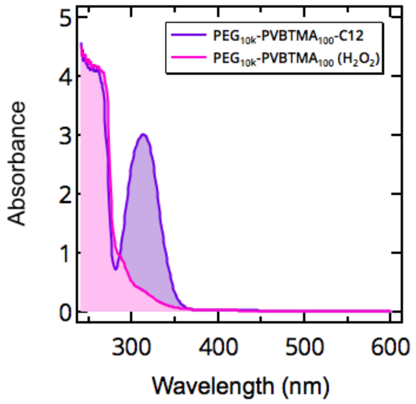

Figure S5. UV-vis spectroscopy of the PVBTMA(**72**)-PEG(10k) system in water before (purple curve) and after (pink curve) introduction of H_2O_2 to remove chain end-groups.

Summary of Polymers and DNA Oligonucleotides. Table S1 shows a summary of the characterized polymers used in this study. Table S2 contains the DNA oligonucleotide sequences.

Table S1. PVBTMA Polymer Characterization.

Polymer	Sample ID	PEO M_n (kg/mol)	Charged Block DP [a]	M_n [b] (kg/mol)	$Đ$ [c]
PVBTMA	PVB50	-	35	7.7	1.12
	PVB100	-	172	36.7	1.09
PVBTMA-PEG	50-1k	1	60	14.2	1.17
	10-5k	5	8	6.6	1.33
	30-5k	5	24	10.0	1.28
	50-5k	5	53	18.3	1.12
	100-5k	5	105	30.9	1.08
	100-10k	10	72	24.4	1.22
	200-10k	10	194	47.7	1.11

[a] Experimentally determined degree of polymerization for the charged blocks. [b] Experimentally-measured absolute molecular weight (M_n), determined by SEC-MALS at 35 °C. [c] Dispersity ($Đ$) = M_w / M_n.

Table S2. DNA Oligonucleotide Sequences.

Length (nt)	Sequence (5′ – 3′)
10	TCAACATCAG
22	CTACCGTCGCATTCAGCATTCA
88	TCAACATCAGTCTGATAAGCTATGGATACTCGTCTGGACTACTTA<br>CTCACTCATTCATCACTATCTACCGTCGCATTCAGCATTCATG

S2. Scattering and Imaging

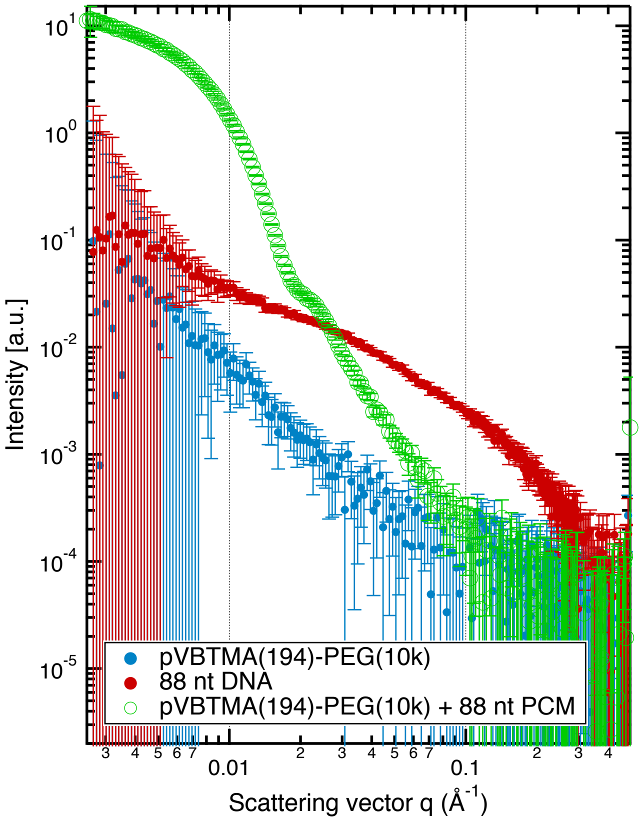

Figure S6. SAXS data for individual polyelectrolytes show no structure formation. Both polyelectrolytes are at 2mM charge concentration in 1xPBS to match experimental conditions. There is no y-axis offset for this plot.

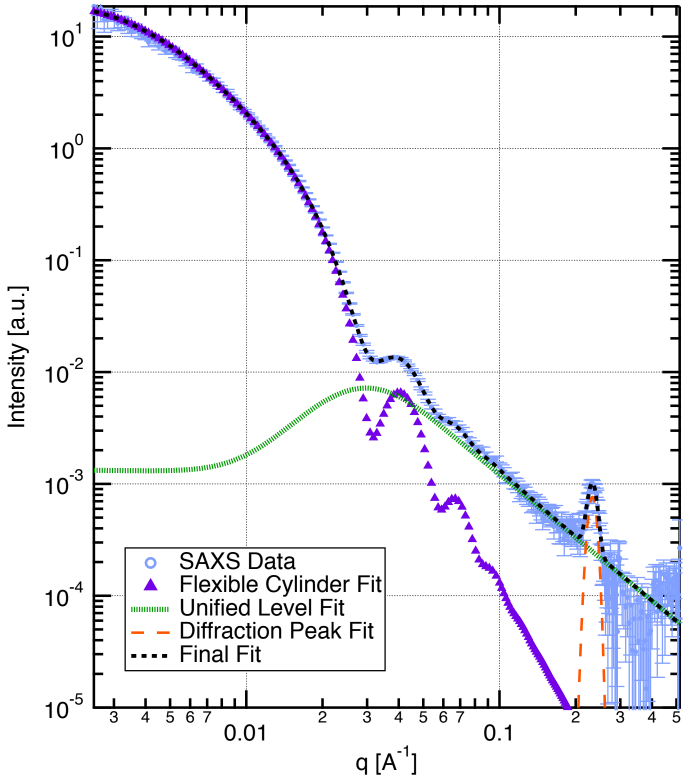

Figure S7. All fitting for SAXS data incorporated the following models: 1) size distribution (purple, above), 2) unified level (green), and 3) diffraction peak (orange), when applicable. Size distribution fits include a shape factor for spheroids, rigid cylinders, or flexible cylinders assuming a Schulz-Zimm distribution and provide micelle shape and size information in mid to low q ranges. A unified level fit is used at high q values ($q \geq \sim 0.1\ A^{-1}$). For data with a high q diffraction peak, this model was added. Original data with error bars is shown in light blue and the final model incorporating all three fits is in black.

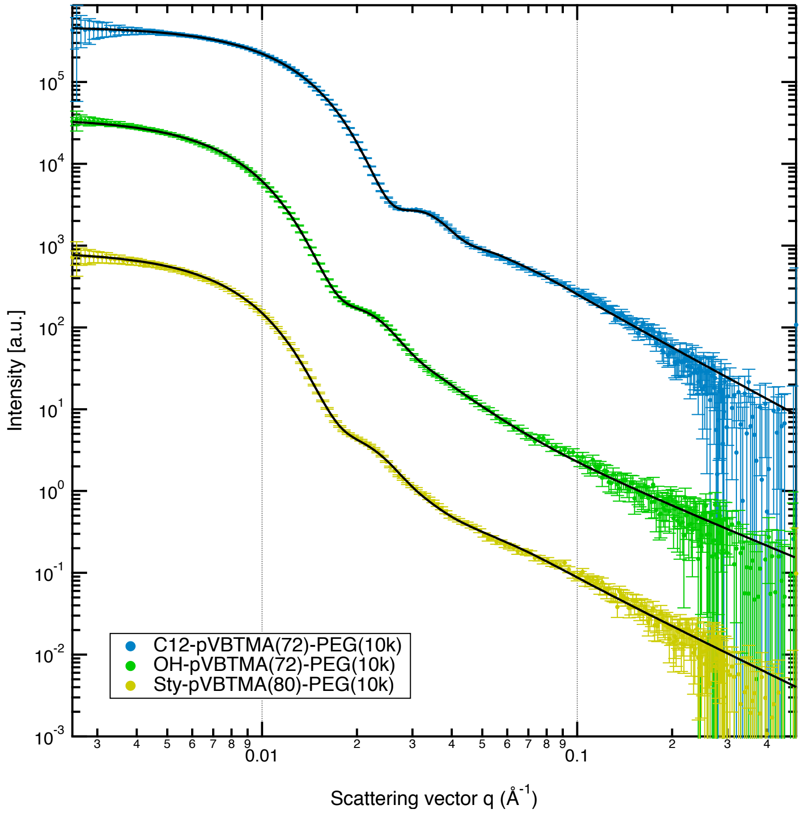

Figure S8. SAXS data to show the effect of PVBTMA end group on PCM size. Styrene and OH end groups appear very similar to each other but larger than the C12 PCMs. 22 nt DNA was used for each sample. Curves are vertically offset for visual clarity.

Table S3: SAXS fitting results for Figure S19 showing the effect of PVBTMA end group on PCM size.

Sample	Mean Radius (nm)	PDI (σ^2/R^2)	Aspect Ratio	Porod exponent
C12-PVBTMA(72)-PEG(10k) + ss22	15.9	0.015	1.4	2.1
OH-PVBTMA(72)-PEG(10k) + ss22	23.5	0.025	1.3	1.6
Sty-PVBTMA(80)-PEG(10k) + ss22	25.0	0.036	1.0	1.9

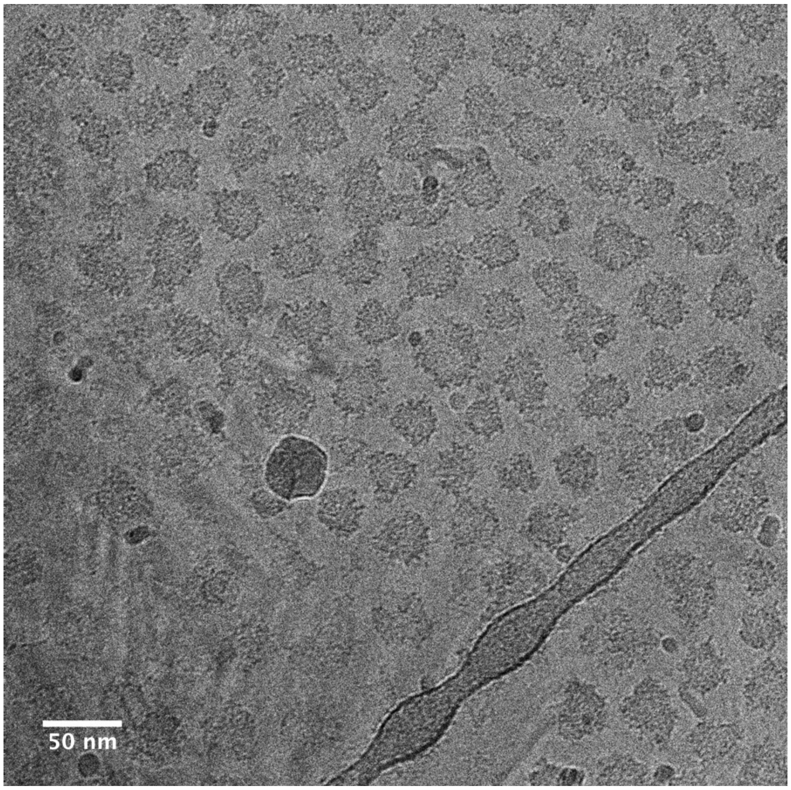

Figure S9. Cryo TEM image of PVBTMA(**72**)-PEG(10k) + 88 nt ssDNA showing spherical micelles. The elongated object in the bottom right is the lacey carbon grid coating.

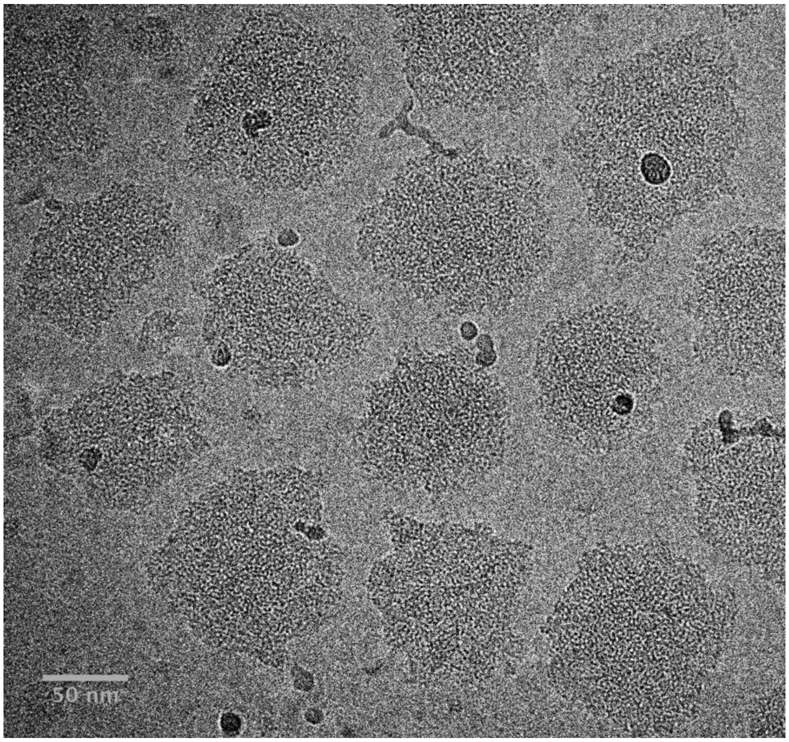

Figure S10. Cryo TEM image of PLys(**200**)-PEG(10k) + 88 nt ssDNA showing spherical micelles.

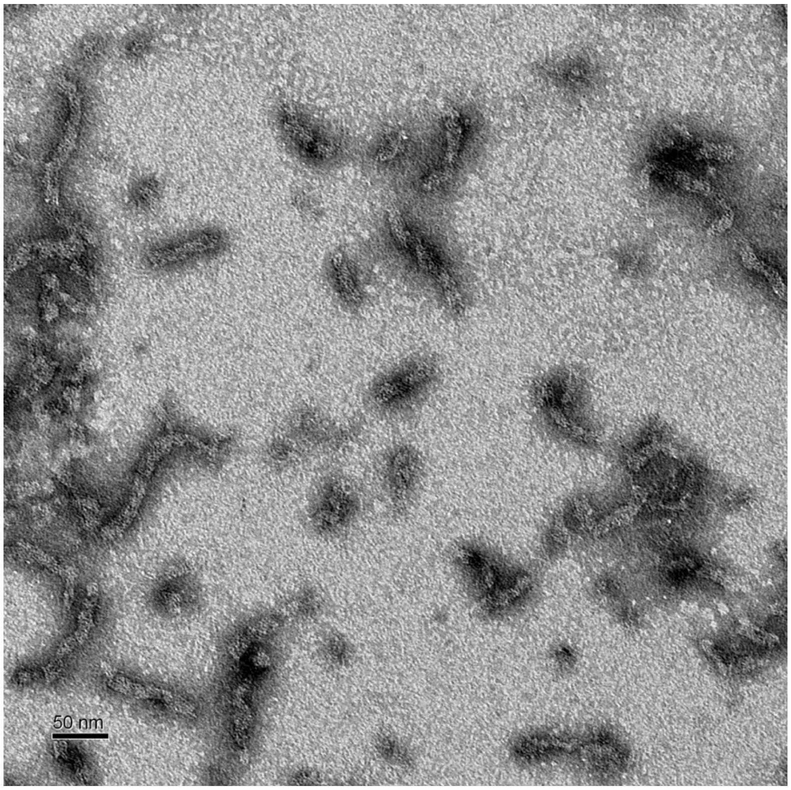

Figure S11. Negative stained TEM image of PVBTMA(**24**)-PEG(5k) + 88 bp dsDNA showing cylinder and worm-like micelles with similar diameters but varying lengths.

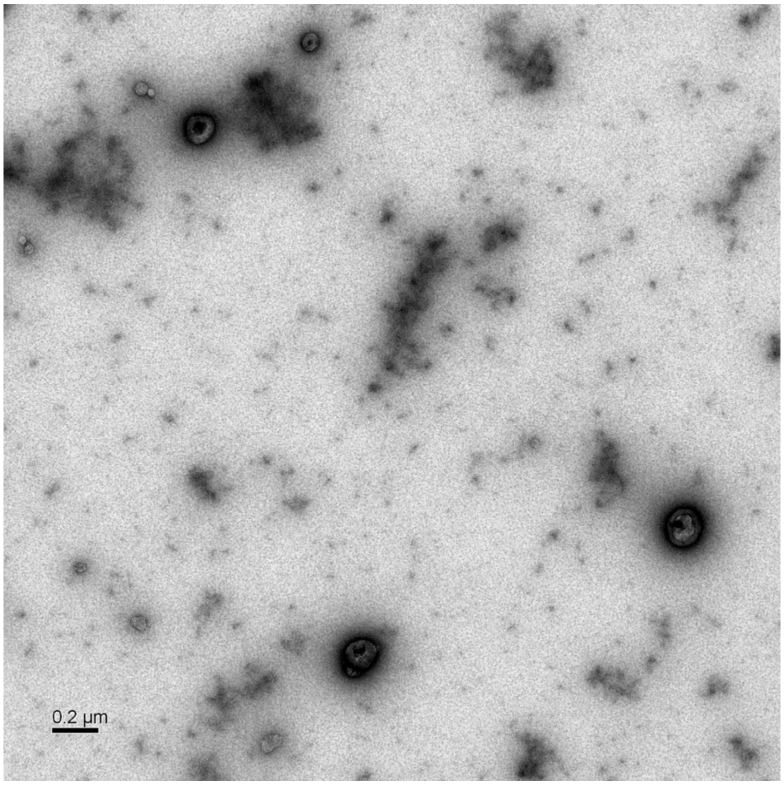

Figure S12. Negative stained TEM image of PVBTMA(**105**)-PEG(5k) + 10 bp dsDNA showing aggregation and a small population of spheroidal micelles.

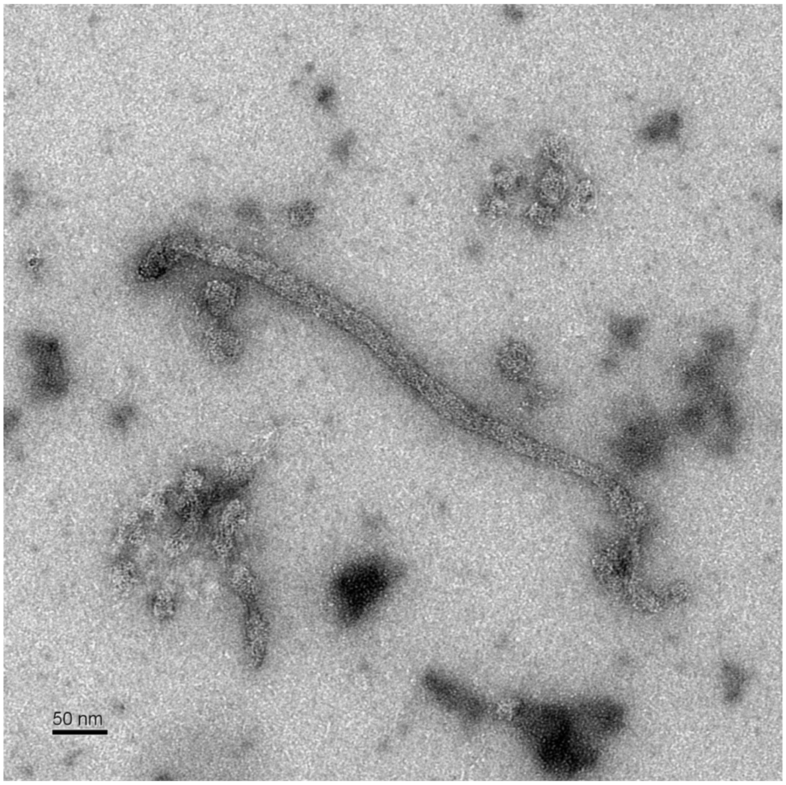

Figure S13. Negative stained TEM image of PVBTMA(**72**)-PEG(10k) + salmon sperm DNA (~2000bp) showing worm-like micelles with similar diameter and varying length.

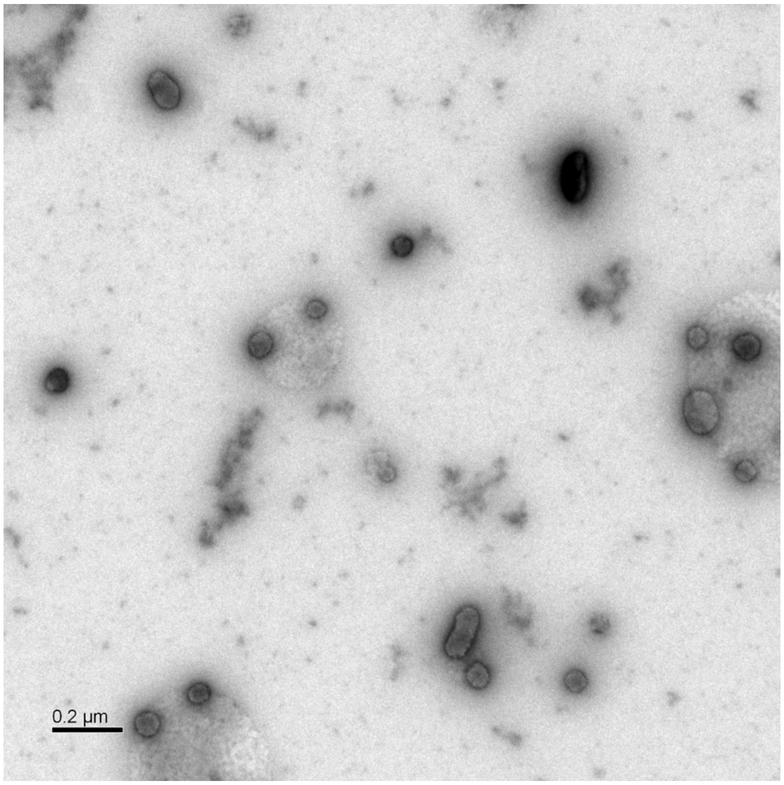

Figure S14. Negative stained TEM image of PVBTMA(**194**)-PEG(10k) + 22 bp dsDNA showing a population of mainly spherical micelles.

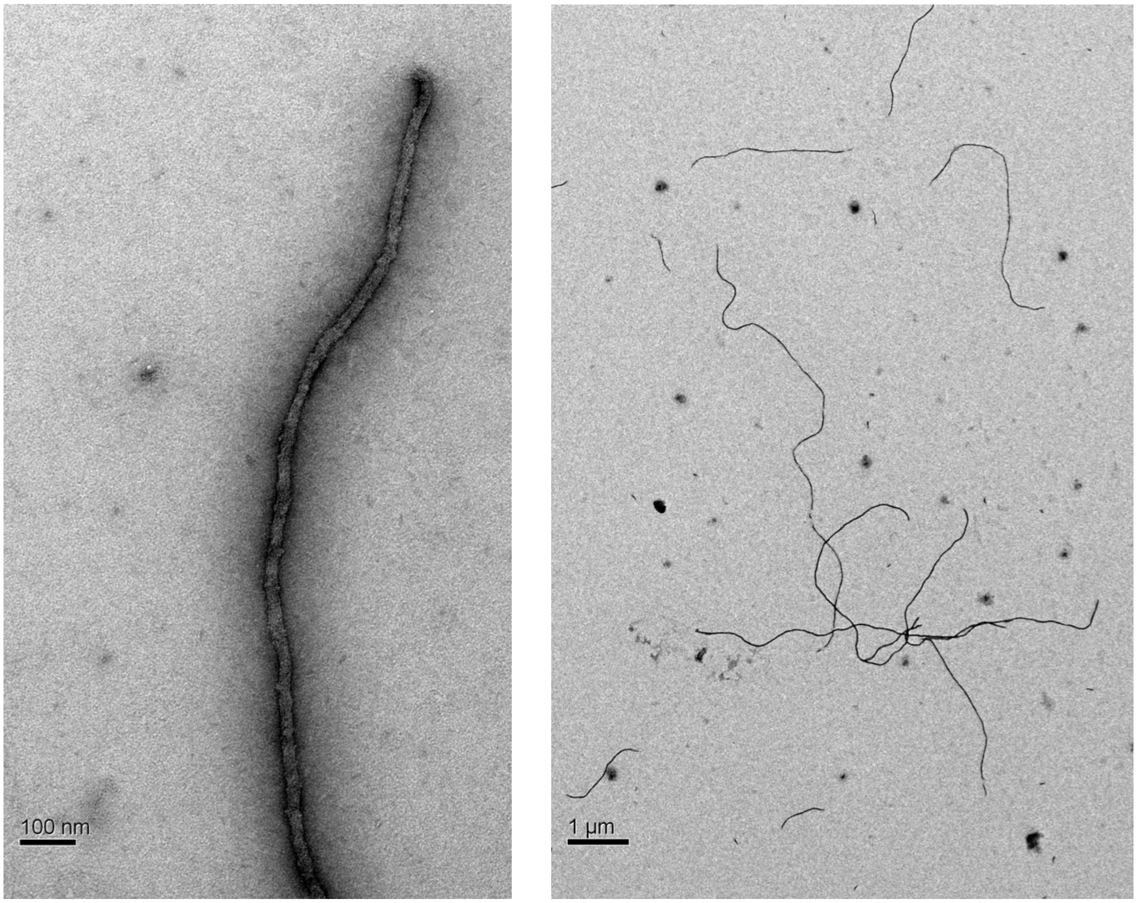

Figure S15. Negative stained TEM images of PLys(**50**)-PEG(5k) + 88 bp dsDNA showing long flexible worm-like micelles.

Table S4: Results of SAXS fitting for PLys-PEG + ssDNA micelles

Sample	Mean Radius (nm)	PDI (σ^2/R^2)	Aspect Ratio	Porod exponent
PLys(**10**)-PEG(5k) + ss22	4.2	0.345	1.0	2.9
PLys(**10**)-PEG(5k) + ss88	4.3	0.007	2.0	2.8
PLys(**30**)-PEG(5k) + ss10	8.8	0.099	1.6	1.8
PLys(**30**)-PEG(5k) + ss22	8.7	0.026	1.6	2.2
PLys(**30**)-PEG(5k) + ss88	7.9	0.027	1.6	2.3
PLys(**50**)-PEG(5k) + ss10	16.0	0.013	1.4	2.3
PLys(**50**)-PEG(5k) + ss22	16.2	0.010	1.4	1.9
PLys(**50**)-PEG(5k) + ss88	15.3	0.018	1.3	2.0
PLys(**100**)-PEG(5k) + ss10	25.6	0.017	1.2	1.9
PLys(**100**)-PEG(5k) + ss22	26.0	0.020	1.1	2.1
PLys(**100**)-PEG(5k) + ss88	23.5	0.000	1.4	1.8
PLys(**100**)-PEG(10k) + ss10	17.7	0.008	1.5	1.8
PLys(**100**)-PEG(10k) + ss22	18.2	0.017	1.5	2.1
PLys(**100**)-PEG(10k) + ss88	16.6	0.014	1.4	1.8
PLys(**200**)-PEG(10k) + ss10	42.5	0.020	1.3	1.5
PLys(**200**)-PEG(10k) + ss22	42.9	0.024	1.1	2.1
PLys(**200**)-PEG(10k) + ss88	41.4	0.016	1.1	1.7
PLys(**10**)-PEG(20k) + ss10	3.1	0.991	1.3	2.1
PLys(**10**)-PEG(20k) + ss22	6.1	0.127	2.5	3.0
PLys(**10**)-PEG(20k) + ss88	2.1	0.692	3.0	2.4
PLys(**50**)-PEG(20k) + ss10	13.0	0.007	1.9	2.6
PLys(**50**)-PEG(20k) + ss22	12.0	0.015	1.9	2.0
PLys(**50**)-PEG(20k) + ss88	11.5	0.017	1.8	2.1
PLys(**100**)-PEG(20k) + ss10	19.1	0.039	2.3	2.2
PLys(**100**)-PEG(20k) + ss22	20.6	0.058	1.5	2.7
PLys(**100**)-PEG(20k) + ss88	19.3	0.013	1.6	2.2

Table S5: Results of SAXS fitting for PVBTMA-PEG + ssDNA micelles

Sample	Mean Radius (nm)	PDI (σ^2/R^2)	Aspect Ratio	Porod exponent
PVBTMA(**24**)-PEG(5k) + ss10	10.1	0.048	1.5	2.0
PVBTMA(**24**)-PEG(5k) + ss22	8.7	0.051	1.6	2.7
PVBTMA(**24**)-PEG(5k) + ss88	8.8	0.031	1.7	1.5
PVBTMA(**53**)-PEG(5k) + ss10	13.1	0.052	3.3	1.7
PVBTMA(**53**)-PEG(5k) + ss22	12.1	0.035	1.9	1.9
PVBTMA(**53**)-PEG(5k) + ss88	12.3	0.024	2.3	1.1
PVBTMA(**72**)-PEG(10k) + ss10	17.7	0.010	1.5	1.6
PVBTMA(**72**)-PEG(10k) + ss22	15.9	0.018	1.4	1.7
PVBTMA(**72**)-PEG(10k) + ss88	14.7	0.020	1.5	1.7
PVBTMA(**194**)-PEG(10k) + ss10	30.0	0.020	1.5	1.6
PVBTMA(**194**)-PEG(10k) + ss22	28.1	0.101	2.0	1.8
PVBTMA(**194**)-PEG(10k) + ss88	22.7	0.015	2.2	3.2

Table S6: Results of SAXS fitting for PLys-PEG + dsDNA micelles

Sample	Mean Radius (nm)	PDI (σ^2/R^2)	Packing Peak	Porod exponent
PLys(**10**)-PEG(5k) + ds22	3.7	0.029	N	2.4
PLys(**10**)-PEG(5k) + ds88	4.0	0.031	Y	2.6
PLys(**10**)-PEG(5k) + salmon	4.6	0.104	Y	1.3
PLys(**30**)-PEG(5k) + ds10	3.8	0.625	Y	3.7
PLys(**30**)-PEG(5k) + ds22	1.8	1.735	Y	2.8
PLys(**30**)-PEG(5k) + ds88	6.4	0.039	Y	2.1
PLys(**50**)-PEG(5k) + ds10	8.6	0.018	Y	2.2
PLys(**50**)-PEG(5k) + ds22	8.8	0.034	Y	2.8
PLys(**50**)-PEG(5k) + ds88	11.0	0.029	Y	1.8
PLys(**50**)-PEG(5k) + salmon	11.4	0.053	Y	1.4
PLys(**100**)-PEG(5k) + ds10	13.7	0.009	Y	1.9
PLys(**100**)-PEG(5k) + ds22	13.5	0.021	Y	2.2
PLys(**100**)-PEG(5k) + ds88	13.9	0.027	Y	1.9
PLys(**100**)-PEG(10k) + ds10	15.1	0.006	Y	1.8
PLys(**100**)-PEG(10k) + ds22	15.2	0.020	Y	2.2
PLys(**100**)-PEG(10k) + ds88	12.2	0.008	Y	1.9
PLys(**100**)-PEG(10k) + salmon	13.3	0.066	Y	2.2
PLys(**200**)-PEG(10k) + ds10	19.9	0.027	Y	2.1
PLys(**200**)-PEG(10k) + ds22	16.3	0.012	Y	2.4
PLys(**200**)-PEG(10k) + ds88	18.1	0.046	Y	1.9
PLys(**10**)-PEG(20k) + ds10	7.0	0.043	N	2.2
PLys(**10**)-PEG(20k) + ds22	7.2	0.022	N	2.5
PLys(**10**)-PEG(20k) + ds88	2.5	0.270	Y	2.3
PLys(**10**)-PEG(20k) + salmon	4.7	0.050	Y	2.5
PLys(**50**)-PEG(20k) + ds10	11.0	0.000	Y	2.0
PLys(**50**)-PEG(20k) + ds22	10.8	0.002	Y	2.3
PLys(**50**)-PEG(20k) + ds88	10.5	0.029	Y	2.3
PLys(**50**)-PEG(20k) + salmon	8.8	0.112	Y	1.6
PLys(**100**)-PEG(20k) + ds10	18.3	0.024	Y	1.9
PLys(**100**)-PEG(20k) + ds22	17.1	0.004	Y	1.9
PLys(**100**)-PEG(20k) + ds88	14.4	0.018	Y	2.0
PLys(**100**)-PEG(20k) + salmon	13.0	0.064	Y	2.8

Table S7: Results of SAXS fitting for PVBTMA-PEG + dsDNA micelles. Low q^x values from data, other parameters from fit.

Sample	Mean Radius (nm)	PDI (σ^2/R^2)	Packing Peak	Porod exponent	Low q^x
PVBTMA(**24**)-PEG(5k) + ds10	10.8	0.043	N	1.5	0.45
PVBTMA(**24**)-PEG(5k) + ds22	10.8	0.044	Y	1.6	0.46
PVBTMA(**24**)-PEG(5k) + ds88	8.6	0.037	Y	1.8	0.96
PVBTMA(**24**)-PEG(5k) + salmon	8.4	0.045	Y	2.2	1.90
PVBTMA(**53**)-PEG(5k) + ds10	14.5	0.060	N	1.3	1.45
PVBTMA(**53**)-PEG(5k) + ds22	12.1	0.088	Y	1.8	1.92
PVBTMA(**53**)-PEG(5k) + ds88	11.0	0.052	Y	1.5	1.13
PVBTMA(**53**)-PEG(5k) + salmon	11.7	0.055	Y	1.4	2.03
PVBTMA(**105**)-PEG(5k) + ds10	14.5	0.029	N	1.2	1.79
PVBTMA(**105**)-PEG(5k) + ds22	15.2	0.038	N	1.4	2.32
PVBTMA(**72**)-PEG(10k) + ds10	18.7	0.018	N	1.2	0.89
PVBTMA(**72**)-PEG(10k) + ds22	19.1	0.019	N	1.6	1.72
PVBTMA(**72**)-PEG(10k) + ds88	15.5	0.052	Y	1.7	1.15
PVBTMA(**72**)-PEG(10k) + salmon	12.7	0.077	Y	2.2	1.99
PVBTMA(**194**)-PEG(10k) + ds10	31.3	0.021	N	1.5	0.65
PVBTMA(**194**)-PEG(10k) + ds22	30.3	0.044	Y	2.0	1.59
PVBTMA(**194**)-PEG(10k) + salmon	17.3	0.046	Y	2.0	2.38

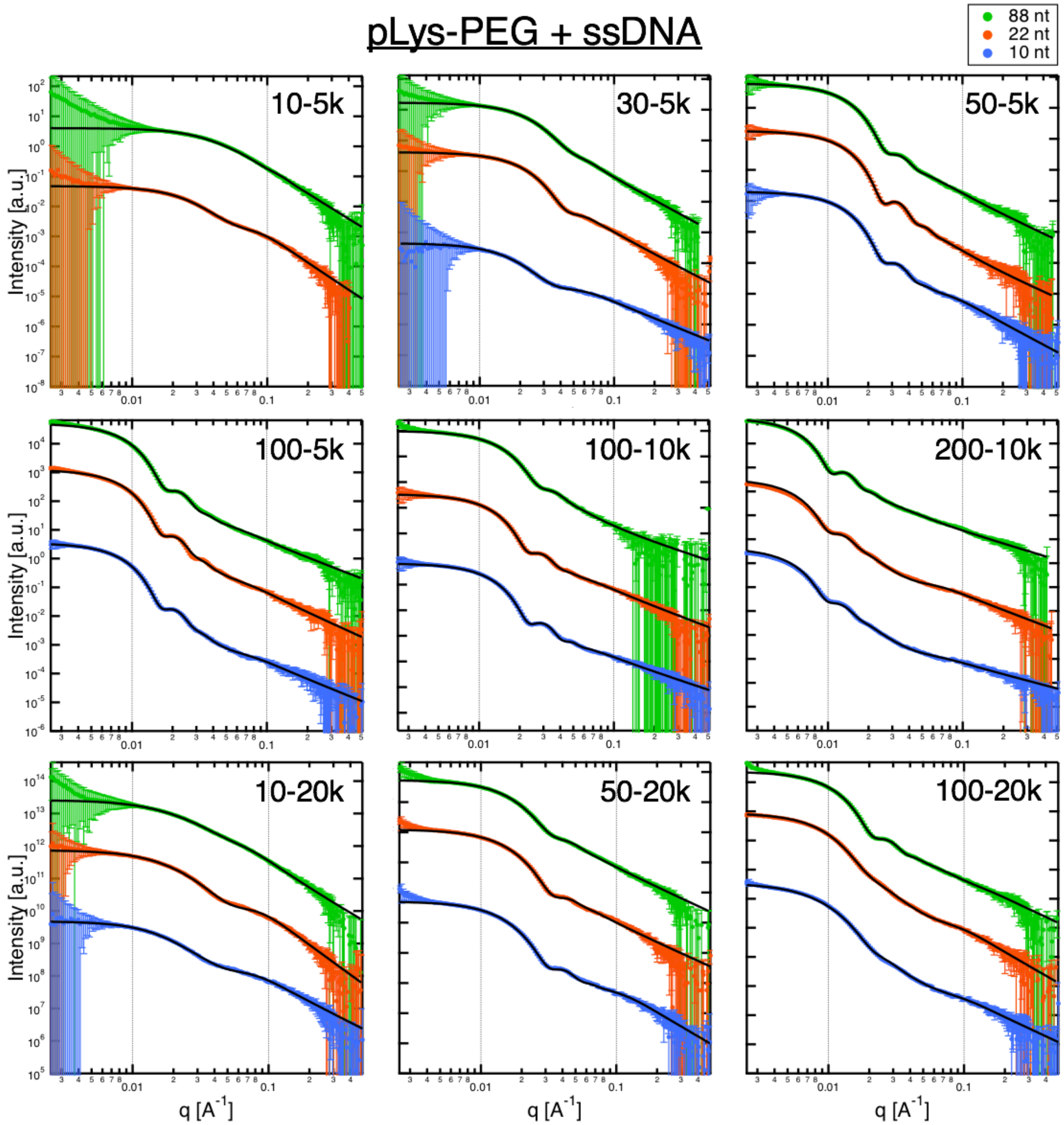

Figure S16. SAXS data and fits for all PLys-PEG and ssDNA micelles. Data is offset in y-axis for clarity.

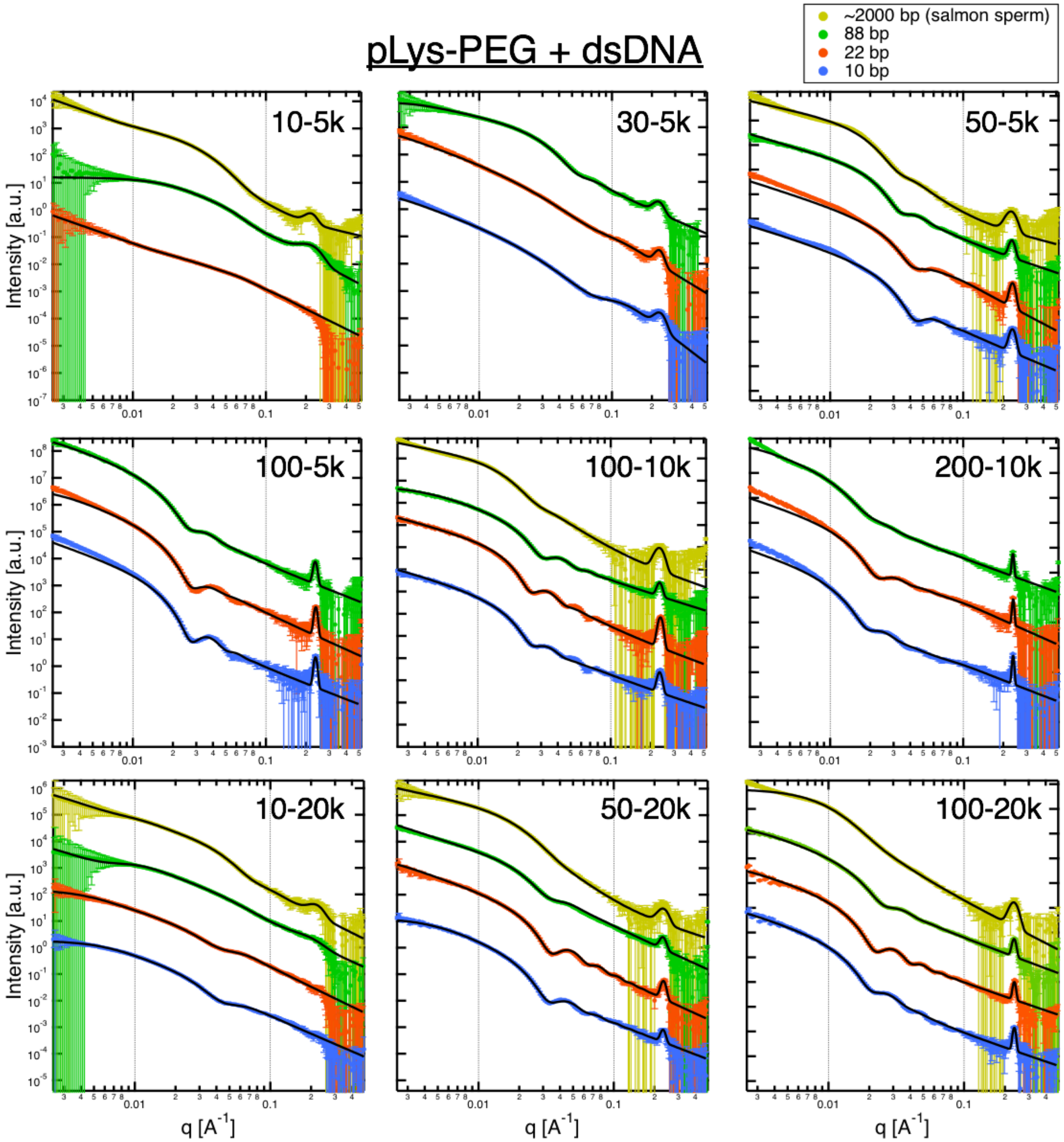

Figure S17. SAXS data and fits for all PLys-PEG and dsDNA micelles. Data is offset in y-axis for clarity.

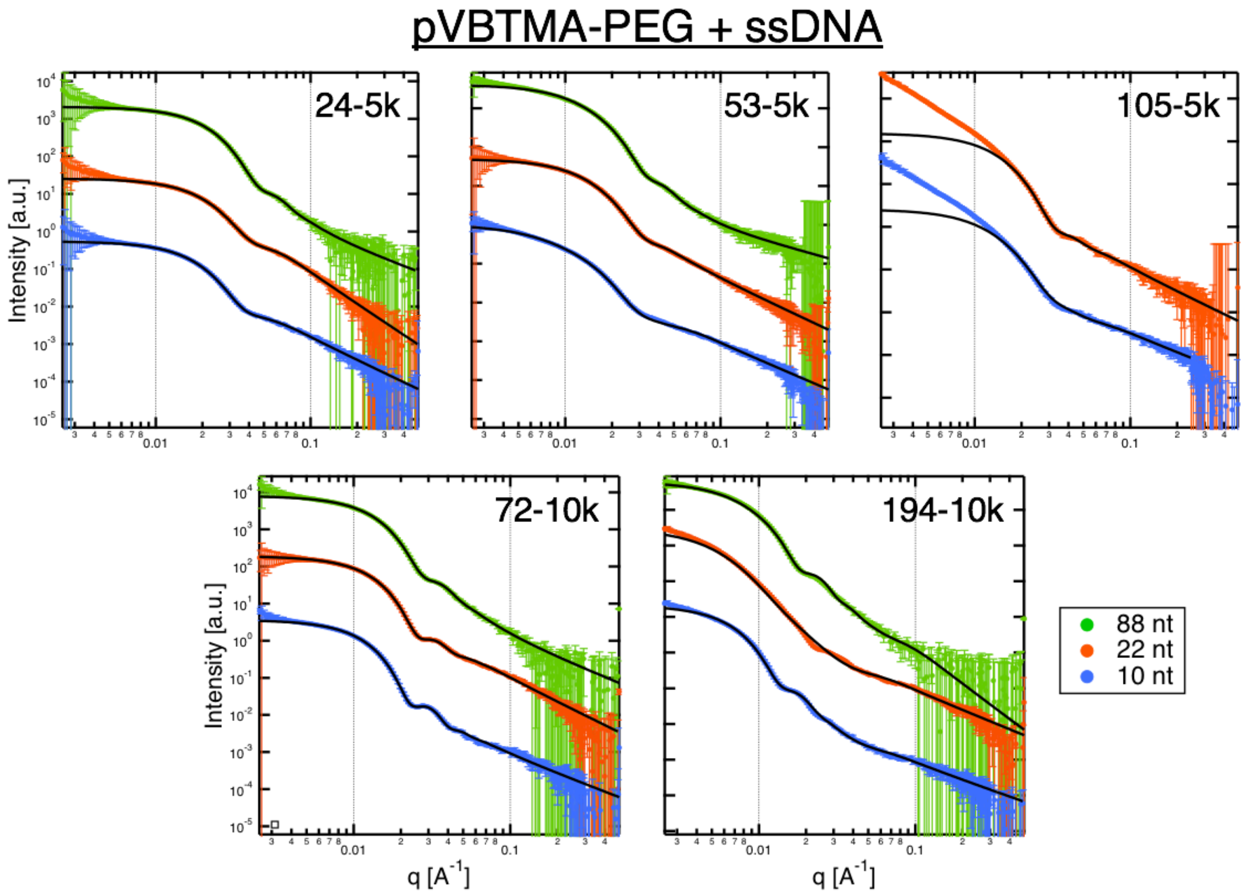

Figure S18. SAXS data and fits for all PVBTMA-PEG and ssDNA micelles. Data is offset in y-axis for clarity.

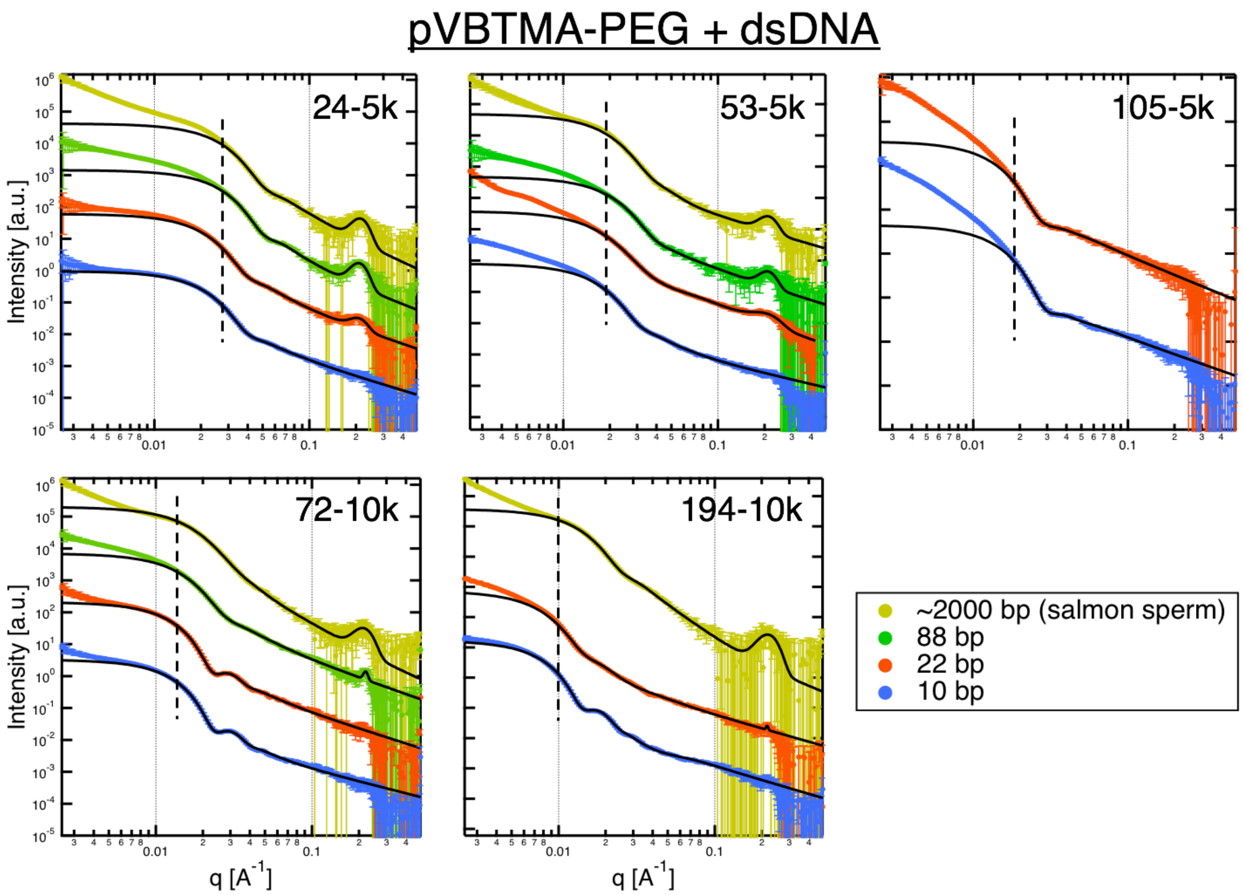

Figure S19. SAXS data and fits for all PVBTMA-PEG and dsDNA micelles. Data is offset in y-axis for clarity. Dotted black lines indicate low q fitting limit for sphere fit.

S3. SI References

1. Ting, J. M.; Wu, H.; Herzog-Arbeitman, A.; Srivastava, S.; Tirrell, M. V. Synthesis and Assembly of Designer Styrenic Diblock Polyelectrolytes. *ACS Macro Lett.* **2018**, *7*, 726–733.
2. Jesson, C. P.; Pearce, C. M.; Simon, H.; Werner, A.; Cunningham, V. J.; Lovett, J. R.; Smallridge, M. J.; Warren, N. J.; Armes, S. P. H_2O_2 Enables Convenient Removal of RAFT End-Groups From Block Copolymer Nano-Objects Prepared via Polymerization-Induced Self-Assembly in Water. *Macromolecules* **2016**, *50*, 182–191.
